# A comprehensive characterization of longevity and culling reasons in Canadian Holstein cattle based on various systematic factors

**DOI:** 10.1093/tas/txad102

**Published:** 2023-08-28

**Authors:** Taiana Cortez de Souza, Luis Fernando Batista Pinto, Valdecy Aparecida Rocha da Cruz, Hinayah Rojas de Oliveira, Victor Breno Pedrosa, Gerson A Oliveira, Filippo Miglior, Flávio S Schenkel, Luiz F Brito

**Affiliations:** Department of Animal Sciences, Federal University of Bahia, Salvador, BA, Brazil; Department of Animal Sciences, Purdue University, West Lafayette, IN, USA; Department of Animal Sciences, Federal University of Bahia, Salvador, BA, Brazil; Department of Animal Sciences, Federal University of Bahia, Salvador, BA, Brazil; Department of Animal Sciences, Purdue University, West Lafayette, IN, USA; Centre for Genetic Improvement of Livestock, Department of Animal Biosciences, University of Guelph, Guelph, ON, Canada; Department of Animal Sciences, Purdue University, West Lafayette, IN, USA; Centre for Genetic Improvement of Livestock, Department of Animal Biosciences, University of Guelph, Guelph, ON, Canada; Centre for Genetic Improvement of Livestock, Department of Animal Biosciences, University of Guelph, Guelph, ON, Canada; Lactanet Canada, Guelph, ON, Canada; Centre for Genetic Improvement of Livestock, Department of Animal Biosciences, University of Guelph, Guelph, ON, Canada; Department of Animal Sciences, Purdue University, West Lafayette, IN, USA; Centre for Genetic Improvement of Livestock, Department of Animal Biosciences, University of Guelph, Guelph, ON, Canada

**Keywords:** dairy cow, durability, herd replacement, productive life, reproductive failure, stayability

## Abstract

The decision of premature culling cows directly impacts the profitability of dairy farms. A comprehensive characterization of the primary causes of culling reasons would greatly improve both management and selection objectives in dairy cattle breeding programs. Therefore, this study aimed to analyze the temporal frequencies of 34 culling reasons in Canadian Holstein cows. After data editing and quality control, records from 3,096,872 cows culled from 9,683 herds spread across Canada were used for the analyses covering the periods from 1996 to 2020. Reproductive issues were the main culling reason accounting for 23.02%, followed by milk production (20.82%), health (20.39%), conformation problems (13.69%), economic factors (13.10%), accidents (5.67%), age-related causes (1.67%), and workability (1.63%). Nearly fifty-eight percent of cows were culled after 47 months of age. The observed frequencies of culling due to economic factors were lower than expected from 1996 to 2014 and higher than expected between 2015 and 2020. Reproduction issues had the highest culling frequencies during fall (24.54%), winter (24.02%), and spring (22.51%), while health issues were the most frequent (22.51%) culling reason in the summer season. Health issues (25.50%) and milk production (27.71%) were the most frequent culling reasons in the provinces of Quebec and Ontario, respectively. Reproductive issues showed the highest frequency across climates based on the Köppen climate classification, except for Csb (Dry-summer subtropical or Mediterranean climate) and Bsk (Middle latitude steppe climate), which correspond to small regions in Canada, where production was the most frequent culling reason (29.42% and 21.56%, respectively). Reproductive and milk performance issues were the two main culling reasons in most ecozones, except in Boreal Shield and Atlantic Marine, where health issues had the highest frequencies (25.12 and 23.75%, respectively). These results will contribute to improving management practices and selective decisions to reduce involuntary culling of Holstein cows.

## Teaser

A detailed characterization of the primary causes of culling reasons in dairy cattle is paramount for improving management and the definition of longevity traits for refining selection indexes in dairy cattle breeding programs. In this study, we investigated the temporal frequencies of 34 culling reasons in Canadian Holstein cows and analyzed the main factors associated with their incidence.

## INTRODUCTION

Factors such as low milk productivity, disease, and infertility can lead to the premature culling of dairy cows (De Vries and Marcondes, 2020). Culling reasons can be classified as either voluntary or involuntary (Zhang et al., 2016). Voluntary culling occurs when there are no apparent problem-related reasons for a breeder to decide to cull her/his cow while diseases, reproductive issues, low milk productivity, and poor conformation were some of the reasons classified as involuntary culling (Fetrow et al., 2006). Involuntary culling can be associated with increased production costs (Zhang et al., 2016). and reducing this type of culling can increase dairy farm profitability. Moreover, higher culling rates also have an environmental impact as additional heifers need to be raised for replacement, and the amount of milk per methane unit will be reduced (i.e., lower environmental efficiency).

The cow’s replacement is an important production cost in dairy farms (Fetrow et al., 2006). Lactanet Canada has reported a cost of CAD $2,650 for raising a 24-month heifer (Fleming and Van Doormaal, 2019). In Great Britain, one-quarter of the dairy farmers recovered the inherent costs of rearing heifers after their first lactation, while the remaining 68% recovered these costs during the second lactation of the cows (Boulton et al., 2017). Grandl et al. (2019) also observed that about 70% of primiparous cows were unable to pay production costs. Moreover, multiparous cows had an average daily milk yield (38.85 kg/day) higher than primiparous cows (30.93 kg/day) (Marumo et al., 2022). Therefore, mature cows are the ones that can pay their rearing cost from birth to calving and generate a financial return for the dairy farmer.

Reproductive issues are among the main causes of premature culling in dairy cows. Intensive genetic selection for milk production contributed to reduced fertility rates and increased incidence of reproductive diseases in dairy cows (Pryce et al., 2004), which is a trend that is now been reverted especially with the more recent implementation of genomic selection for fertility and reproduction traits (Ma et al., 2019; Brito et al., 2021). Inadequate body condition can also lead to issues in dairy cows during lactation, including increased susceptibility to metabolic disorders and compromised fertility (Weigel, 2006). The main reproductive disorders in dairy cows are metritis, abortion, placental retention, dystocia, and uterine diseases (Abdisa, 2018; Guarini et al., 2019). One example of such reproductive-related diseases is metritis, which negatively impacts milk production (Dervishe et al., 2016) and animal welfare. Mastitis (Martin et al., 2018), leg and foot disorders, and metabolic and respiratory diseases (Hu et al., 2021) are also listed among the main diseases affecting the longevity of dairy cows. Despite the advances in managing mastitis, it remains the costliest bacterial disease of dairy cattle (Ruegg, 2017) and the major disease recorded in dairy farms (Koeck et al., 2012; Martin et al., 2018). The presence of mastitis associated with reduction in milk production, followed by culling costs and preventive actions (Aghamohammadi et al., 2018), in addition to greater antibiotics usage. Clinical mastitis has been reported as one of the main risk factors for early culling in Quebec dairy herds (Haine et al., 2017). Metabolic disease is another crucial factor of premature culling, as it is associated with a reduction in milk production and compromised reproductive performance (Paiano et al., 2019).

Approximately 32% of dairy cows are culled annually in Canada and a large proportion of this is due to involuntary culling, 37.4% for reproductive reasons, and 21.46% for mastitis (CDIC, 2021a). Lactanet Canada (Guelph, ON and St Anne de Bellevue, QC, Canada), a not-for-profit farmer-owned organization serving Canadian dairy industry and producers, has recorded the culling reasons of dairy cows in Canada since the 20th century, but this database has not yet been comprehensively evaluated with respect to all major causes of culling in Holstein cattle. The analysis of this database can guide effective management actions for reducing early involuntary culling of dairy cows and perhaps refining current breeding goals. For instance, recent studies in North American Angus cattle reported different variance components and heritabilities estimated for longevity depending on the culling reason (Oliveira et al., 2020a, 2020b). Thus, the main objectives of this study were to describe the frequencies of different culling reasons in Canadian Holstein cows and evaluate the impact of systematic factors such as cow age, year, season, province, climate, and ecozone on the frequencies of different culling reasons.

## MATERIALS AND METHODS

### Ethics Statement

The present study was conducted using existing phenotypic and pedigree information from the Lactanet (www.lactanet.ca; Guelph, ON, and St Anne de Bellevue, QC, Canada) database. Therefore, no animal experiments were carried out and the approval of the animal care committee was not needed.

### Dataset and Quality Control

The present study analyzed 7,188,910 culling reason records from Canadian Holstein cows, which were recorded between 1996 and 2021. The following information was available: pedigree, cows’ birth date, the dates of entry and exit of each cow in a herd, cows’ culling reason, herd location, and period of the year in which the cows were culled off. Data quality control was performed keeping information that passed the following criteria: 1) cows with date records for both entry and exit from the herds where they were raised, 2) herds with culling reasons recorded over the years (for this filter, a pedigree going back at least three known generations in the same herd was used—to avoid including herds that recorded information for a short period), 3) Cows with age between 12 and 180 months (to avoid very young heifers and cows with age far above the generation interval), 4) the cows that were not born in the same herd in which they had their culling reason recorded in the database, given that their date of entry in the culling herd occurred up to 24 months of age, ensuring that their first lactation occurred at least partially in the culling herd, and 5) cullings recorded before 1996 were excluded because the frequency of annotation before that year was limited.

After the data edition, 3,096,872 cows from 9,683 herds remained in the dataset, being 704,145 with unknown culling reasons and 2,392,727 with known culling reason records evaluating in the periods from 1996 to 2020 (Supplementary Table 1). A total of 34 culling reasons were kept, the culling reason classes “Unknown” and “Other” were grouped as “Missing”. The other 32 culling reasons were grouped into eight categories by merging less frequent culling reasons with others of higher frequencies. Some cows could have been culled due to multiple reasons, but only the main one (recorded by the farmer) was considered for the study. The final culling reason categories were defined as follows: 1) accident (injury to udder/teats, injury, poison, and electrocution), 2) economic factors (rented out, exported, transferred, herd off test, and sold for veal), 3) age-related causes, 4) production performance (dairy production, low milk production, low milk fat, and low milk protein), 5) reproductive performance (reproductive and calving difficult), 6) workability (bad temperament and slow milker), 7) conformation (overall conformation, feet and legs, and udder conformation), and 8) health (mastitis, milk fever, Johne’s disease, leukosis, peritonitis, pneumonia, displaced abomasum, arthritis, sickness, *Staphylococcus aureus*, and bloat). The cows were raised in herds located across all provinces in Canada. Six different climatic regions were defined based on the Köppen climate classification, including BSk = Middle Latitude Steppe climate (Cold semi-arid), Cfb = Marine West Coast climate (Oceanic), Csb = Subtropical or Mediterranean climate (dry and cool summer), Dfa = Humid and hot summer Continental climate, Dfb = Humid and cool summer Continental climate, Dfc = Subarctic climate, and ET = Tundra climate (Government of Canada, 2022). Moreover, seven different terrestrial ecozones were found in this final dataset, including Atlantic Maritime, Boreal Plain, Boreal Shield, Mixed Wood Plain, Montane Cordillera, Pacific Maritime, and Prairie (Statistics Canada, 2017). The culling seasons were defined as: spring (from March 20 to June 20), summer (from June 21 to September 22), fall (from September 23 to December 20), and winter (from December 21 to March 19).

### Statistical Analyses

The Chi-square test was used to assess the effects of the factors (cow’s age, year, season, province, climate, and ecozone) on the frequencies of the eight culling reason groups (i.e., accident, economic, age-related, production, reproduction, workability, conformation, and health). This was done by comparing the observed versus expected culling frequencies in each level of the factor analyzed. A significance level of 5% was used in all tests. All data editing quality control and preparation of tables and figures were done using the R software (R Core Team, 2022).

## RESULTS

### Distribution of Culling Reasons by Cow Age

Reproduction was the main cause of culling in Canadian Holsteins with 23.02% of frequency, followed by milk production (20.82%), health issues (20.39%), conformation problems (13.69%), economic reasons (13.10%), accidents (5.67%), age-related culling (1.67%), and workability (1.63%) (Table 1). Most of the culling occurred on animals 48 months or older, with a total of 58.09%. The economic culling reason was higher on animals between 12 and 23 months of age, accounting for 41.33% of the total culling records at this age period, while milk production was the main culling reason (33.36%) on animals between 24 and 35 months of age. From 36 months of age and older, health and reproductive issues became the main culling reasons, except in cows older than 120 months, where age-related culling exceeded both health and reproductive issues. The observed frequency of culling due to economic reasons between 12 and 23 months was higher than expected (observed = 81,383 and expected = 25,804) (*P* < 0.0001). Moreover, the culling frequency of cows between 24 and 35 months of age due to health issues were higher than expected (observed = 172,390 and expected = 107,554) (*P* < 0.0001).

**Table 1.** Distribution of frequencies of the eight culling reasons by age classes in Canadian Holstein cows.

Culling reasons	Age at culling (in months)										Overall frequencies	
	12-23	24-35	36-47	48-59	60-71	72-83	84-95	96-107	108-119	≥120		
Reproduction	32,596	79,255	126,846	116,169	81,573	52,897	31,339	16,094	8,034	6,038	550,841	23.02%
Production	52,764	172,390	105,666	69,276	44,261	26,637	14,532	7,007	3,228	2,316	498,077	20.82%
Health	10,270	77,784	98,185	103,345	84,059	55,954	31,701	15,540	6,883	4,213	487,934	20.39%
Conformation	9,708	58,166	59,841	63,819	55,615	39,455	22,834	11,094	4,715	2,425	327,672	13.69%
Economic	81,383	76,602	58,913	40,738	25,743	15,028	8,037	3,869	1,783	1,440	313,536	13.10%
Accident	8,253	35,231	28,190	23,489	17,560	11,331	6,294	3,184	1,327	924	135,783	5.67%
Age-related culling	136	456	776	1,737	3,859	6,246	7,565	6,874	5,295	7,010	39,954	1.67%
Workability	1,813	16,798	7,726	5,321	3,536	2,022	1,037	432	157	88	38,930	1.63%
Overall frequencies	196,923	516,682	486,143	423,894	316,206	209,570	123,339	64,094	31,422	24,454	2,392,727	100%
	8.23%	21.59%	20.32%	17.72%	13.22%	8.76%	5.15%	2.68%	1.31%	1.02%		

### Distribution of Culling Reasons by Year

**Figure 1.**
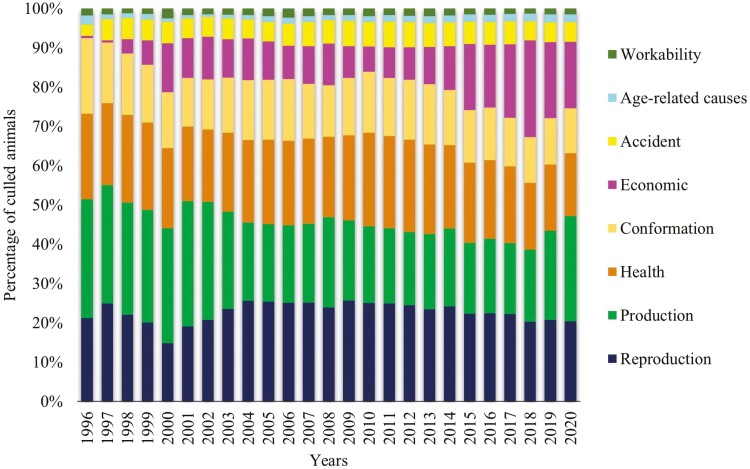
Distribution of culling reasons by year in Canadian Holstein cows.

The total number of culling reason records increased from 1996 (2,329 records) to 2020 (164,794 records) (Supplementary Table 1). Economic reasons have become one of the main culling reasons since 2015 (Figure 1). A Chi-square test to compare the period from 1996 to 2014 vs. 2015 to 2020 was performed and a significant (*P* < 0.0001) difference was observed between the periods. From 1996 to 2014, the number of cows culled based on economic reasons was lower than expected (observed = 128,222 and expected = 184,889). On the other hand, the number of cows culled due to economic reasons from 2015 to 2020 was higher than expected (observed = 185,314 and expected = 128,647) (*P* < 0.0001). As depicted in Figure 1, after the implementation of genomic selection in Canada in 2009, the frequency of culling based on health, reproduction, and conformation problems decreased, while culling based on economic reasons increased (Figure 1).

### Distribution of Culling Reasons by Season

**Figure 2.**
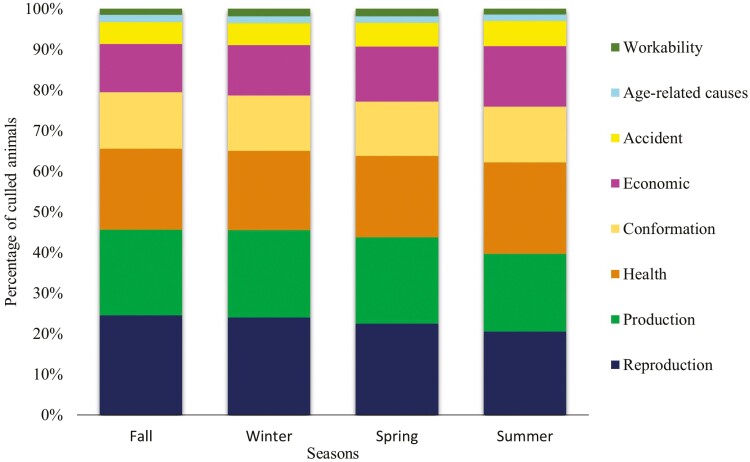
Distribution of culling reasons by season in Canadian Holstein cows.

Winter and fall had culling frequencies of 27.30% and 26.65%, respectively, which were slightly higher than the frequencies observed in the spring (23.72%) and summer (22.33%) seasons (Supplementary Table 2). Reproduction issues were the highest culling frequencies in the fall (24.54%), winter (24.02%), and spring (22.51%), while health issues were the major reason (22.51%) in the summer (Figure 2). In the summer, the number of cows culled due to both milk production (observed = 102,197; expected = 111,198) and reproduction (observed = 109,615; expected = 122,977) issues were below expected (*P* < 0.0001), while conformation (observed = 79,436 and expected = 69,998) and health (observed = 120,259 and expected = 108,933) issues were higher than expected in the summer season (*P* < 0.0001).

### Distribution of Culling Reasons by Province

**Figure 3.**
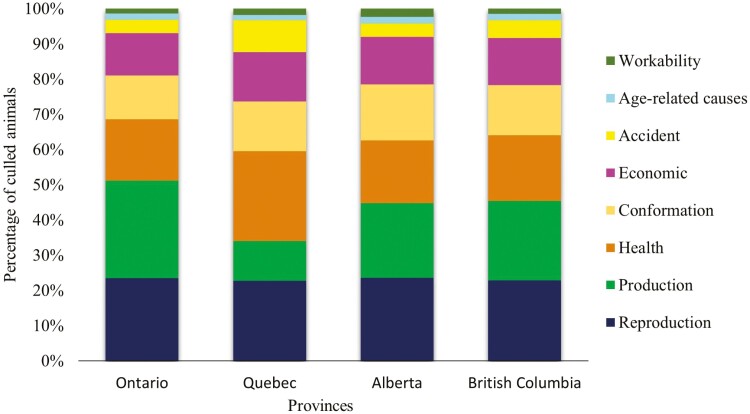
Distribution of culling reasons in the four main dairy production provinces in Canada.

Distribution of culling frequencies of Canadian Holstein cows by provinces were also analyzed (Table 2 and Supplementary Table 3). As expected, Quebec, Ontario, Alberta, and British Columbia concentrated the largest number of culling records (2,135,824), i.e., 89.26% of the total, as they are the largest dairy cattle producers in Canada. Quebec had 37.0% of culling records and 4,548 farms, followed by Ontario with 32.1% and 3,298 farms, Alberta with 8.9% and 488 farms, and British Columbia with 8.9% and 461 farms in 2020-2021 (CDIC, 2021b, 2021c). Quebec had health issues as the highest culling frequency (25.00%), while Ontario had milk production (27.75%) as the primary culling reason (Figure 3). The Chi-square test indicated a significant effect of the province on the frequencies of different culling reasons (*P* < 0.0001). In Ontario, the number of cows culled based on health issues (observed = 166,215 and expected = 194,091), accidents (observed = 36,154 and expected = 54,012), and conformation issues (observed = 118,406 and expected = 130,342) were lower than expected; while the frequency of culling due to milk production was higher than expected (observed = 263,767 and expected = 198,125) (*P* < 0.0001). In Quebec, the frequencies of culling due to health issues (observed = 182,164 and expected = 145,691) and accidents (observed = 64,785 and expected = 40,543) were higher than expected; while the frequency of culling due to milk production was lower than expected (observed = 80,985 and expected = 148,720) (*P* < 0.0001).

**Table 2.** Distribution of frequencies of the eight culling reasons by herd province in Canadian Holstein cows.

Culling reasons	Province									
	Ontario	Quebec	Alberta	British Columbia	Manitoba	Saskatchewan	New Brunswick	Nova Scotia	Prince Edward Island	Newfoundland and Labrador
Reproduction	223,594	162,685	58,635	50,575	22,443	12,339	7,263	6,885	5,756	666
Production	263,767	80,985	52,758	49,898	16,596	13,850	6,138	7,035	6,463	587
Health	166,215	182,164	44,064	41,424	20,736	12,817	6,995	6,921	5,951	647
Conformation	118,406	100,926	39,683	31,372	15,452	9,274	4,388	4,506	3,351	314
Economic	113,762	100,025	33,567	29,729	13,282	10,969	4,448	4,324	3,315	115
Accident	36,154	64,785	9,227	11,201	4,301	3,412	2,632	2,458	1,413	200
Age-related culling	16,936	10,227	4,898	3,804	1,678	1,116	500	322	429	44
Workability	12,946	12,642	5,558	3,212	2,114	1,156	492	433	310	67
Overall frequencies	951,780	714,439	248,390	221,215	96,602	64,933	32,856	32,884	26,988	2,640
	39.78%	29.86%	10.38%	9.25%	4.04%	2.71%	1.37%	1.37%	1.13%	0.11%

### Distribution of Culling Reasons by Climatic Region

**Figure 4.**
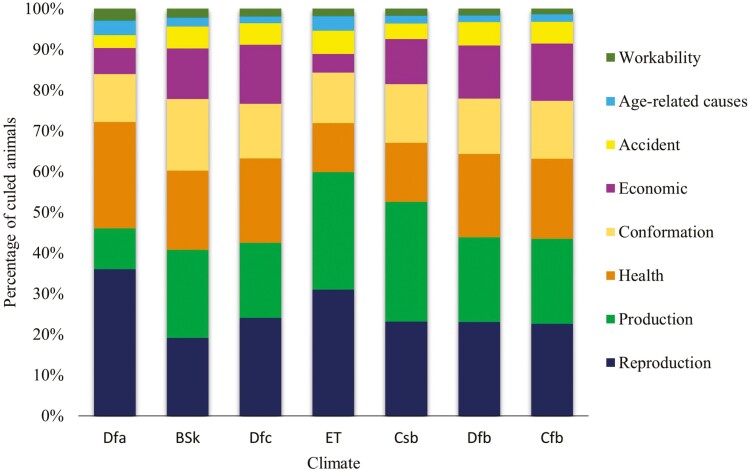
Distribution of culling reasons by climatic regions in Canada (BSk = Middle Latitude Steppe climate (Cold semi-arid), Cfb = Marine West Coast climate (Oceanic), Csb = Subtropical or Mediterranean climate (dry and cool summer), Dfa = Humid and hot summer Continental climate, Dfb = Humid and cool summer Continental climate, Dfc = Subarctic climate, and ET = Tundra climate).

The cows were raised across seven climatic regions (Figure 4), with 2,105,755 records (88.01%) on Dfb (Humid and cool summer Continental climate) (Supplementary Table 4). Reproductive issues were the main culling reason across climates (Figure 4), except in Csb (Subtropical or Mediterranean climate) and BSk (Middle Latitude Steppe climate), where milk production was the major culling reason (29.42% and 21.56%, respectively). Culling due to reproductive issues showed an interesting variation among climates, with higher frequencies in Dfa (Humid and cool summer Continental climate) (36.07%) and lower frequencies in BSk (19.18%). Health culling frequency (26.07%) was especially high in the Dfa climate. The climate was another significant factor affecting culling frequencies (*P* < 0.0001). The highest differences between observed and expected frequencies were found for milk production (observed = 6,889 and expected = 4,875) and health issues (observed = 3,409 and expected = 4,775) in the Csb climate.

### Distributions of Culling Reasons by Ecozone

**Figure 5.**
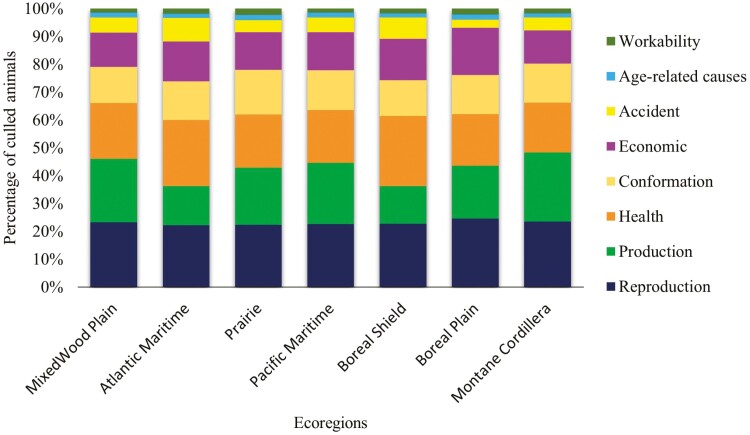
Distribution of culling reasons by ecoregions in Canadian Holstein cows.

Cows from seven ecozones in Canada were analyzed (Figure 5). The MixedWood Plain was the predominant ecozone with 1,341,826 cows (56.08%) (Supplementary Table 5). Reproduction and milk production were the two major culling reasons in the MixedWood Plain (23.32% and 22.85%, respectively). These culling reasons were also the main ones in other regions, except in the Boreal Shield and Atlantic Marine, where health-based culling had the highest frequencies (25.12% and 23.75%, respectively). The ecozones had a significant (*P* < 0.0001) effect on the frequencies of culling. In Atlantic Maritime, culling based on milk production was lower than expected (observed = 44,136 and expected = 65,309), while the amount of culling due to an accident (observed = 26,576) and (expected = 17,804) or health (observed = 74,503 and expected = 63,979) was higher than expected. In MixedWood Plain, culling due to milk production was higher than expected (observed = 306,641 and expected = 279,318). In Boreal Shield, the culling frequency due to milk production was lower than expected (observed = 14,548 and expected = 22,505), while the frequency due to health issues was higher than expected (observed = 27,158 and expected = 22,047).

## DISCUSSION

Previous studies have investigated mortality and culling reasons in dairy cattle (Pinedo et al., 2010; Heise et al., 2016; Zhang et al., 2019). However, few studies were based on such a large number of cows with known culling reasons across so many years. In this study, the frequencies of 34 culling reasons were calculated using a database of more than 2.3 million Holstein cows culled across 25 years (1996 to 2020). Then, the culling reasons were grouped into eight groups of main culling reasons for further analyses. The effects of six systematic factors (i.e., year and season of culling, cow age, province, climate, and ecozone) on the frequencies of culling reasons were evaluated. Reproduction, health, and milk production were the main culling reasons observed in Canada during the evaluated period. O’Bleness and Van Vleck (1962) and Pinedo et al. (2010) also reported productive and reproductive reasons as the main culling reasons in North American Holstein cows.

The study by VanRaden and Wiggans (1995) has already evaluated the productive life with other traits of dairy cattle for the development of a selection index. After the implementation of genomic selection in Canada in 2009, the frequency of culling based on health, reproduction, and conformation decreased, while culling based on economic reasons increased (Figure 1). This is in agreement with Miglior et al. (2017), who stated that, over time, it can be seen that with inclusion of functional traits in the selection indices and the introduction of genomic selection in Canada, an increase in genetic progress in traits traditionally more difficult to select for has been achieved (Miglior et al., 2017). In addition, the increased use of sexed semen coupled with genomic selection after 2015 (Sweett and Van Doormaal, 2022) is a strategy used to accelerate genetic gains by increasing the number of top replacement heifers, with the excess of heifers being sold. However, herd mortality can affect selection intensity, leading to a reduction in genetic gain over time (VanRaden et al., 2021). Moreover, the use of beef semen in dairy has substantially increased since 2011, aiming to produce crossbred young calves for sale at premium prices (Sweett and Van Doormaal, 2022).

Culling due to reproductive issues was the most frequent cause of cow culling at 23.02%, (Table 1), which indicates the importance of continuing to breed for improved fertility in the Holstein breed. Ahlman et al. (2011) and Adamczyk et al. (2018) reported reproduction culling rates equal to 24.8% and 39.6% in the Swedish and Polish Holstein populations, respectively. The improvement of fertility and reproductive efficiency is a key breeding goal in modern dairy cattle breeding programs as previous studies have reported an unfavorable genetic correlation between milk production and reproductive efficiency indicator traits (Pryce et al., 2004; Miglior et al., 2017; Oliveira-Junior et al., 2021). For instance, there is an unfavorable genetic correlation (0.43 ± 0.01) between milk production and open days (Oliveira-Junior et al., 2021) and the genetic trends for fertility were decreasing in North American Holstein cattle until reproduction traits were added to the selection indices (Miglior et al., 2017; Brito et al., 2021). In Canada, over 15 fertility and reproduction traits have been added to the genetic evaluation schemes (Van Doormaal et al., 2007), including age at first insemination, calving ease, and days from first service to conception (Jamrozik et al., 2006).

Low production was the most frequent culling reason for cows aged between 24 and 35 months of age. In the study on the productive life of cows, Ducrocq (1994) observed that the reasons for culling are not necessarily related to age at first calving, but rather to the stages of lactation and low milk production. This indicates that a significant number of cows, around 50% (Table 1) of the total culled cows, were culled before the third lactation, i.e., before reaching the productive maturity observed around the fifth lactation with highest milk yields (Grandl et al., 2016). In addition to not reaching the productive peak, these cows are also being culled before offsetting their raising and maintenance costs. A cow generates costs to the dairy farm throughout its life, but it is just after calving that it generates revenue from the sale of milk and, eventually, with the sale of its progeny. In Canada, the revenue from cows exceeds their cost when they are around 42 months of age, that is when they are already in their second lactation.

**Figure 6.**
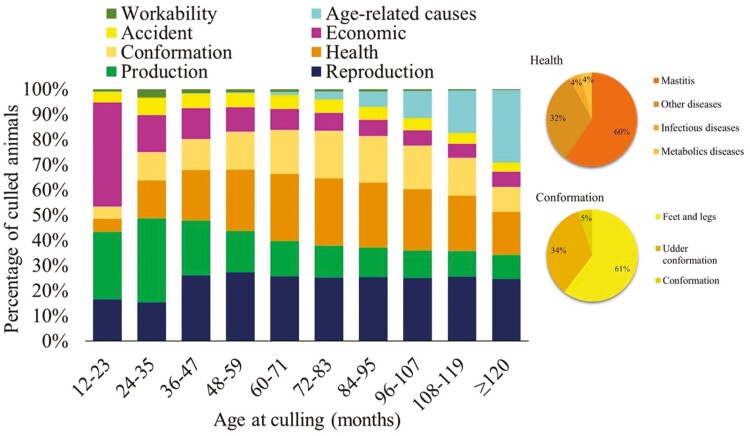
Distribution of culling reasons by age classes in Canadian Holstein cows.

The main health culling reason was mastitis, which accounted for 59.82% of the health culling records (Figure 6). In the last 5 years (2016–2020), mastitis accounted for 10.67% of the total culling reported among herds. Gröhn et al. (1998) also reported mastitis as the main health culling reason. According to Haine et al. (2017), clinical mastitis is a major risk factor for culling dairy cows in Quebec, analyzing herds with at least 10% incidence of clinical mastitis in lactation. Koeck et al. (2012) identified that the highest disease rate occurs in the first month of lactation, and mastitis had the highest prevalence (12.6%) in Canadian dairy herds. This is one of the most frequent and costly infectious diseases in dairy cattle (Halasa et al., 2007). Health problems, especially metabolic and udder diseases, may also be a consequence of the intensive selection for greater milk production. For instance, unfavorable positive genetic correlations have been observed between milk production and somatic cell score in dairy cattle of different breeds (Sabedot et al., 2018; Chegini et al., 2019). Significant decreases in milk production are observed in cows with mastitis (Puerto et al., 2021). In addition, cows with mastitis often have lower pregnancy rates (Ahmadzadeh et al., 2009). Dairy cows usually calve for the first time at around two years of age (De Vries, 2020) and their productive life expectance ranges from 3 to 4.5 years (Kerslake et al., 2018; De Vries and Marcondes, 2020). The results found here show that 42% of the cows are culled between 24 and 47 months of age (Table 1), i.e., way before their productive life expectancy (De Vries and Marcondes, 2020).

Selection to reduce early culling will increase productive life, resulting in improvements in the profitability of the dairy industry, in addition to having greater animal welfare. However, part of this early culling is voluntary. For instance, in the present study, the economic factor was the most frequent (41.32%) culling reason for cows aged between 12 and 23 months (Table 1). Overton and Dhuyvetter (2020) have indicated that advancements in reproductive management and the use of sexed semen have led to an increase in the number of replacement heifers in US dairy herds. According to De Vries and Marcondes (2020), the surplus of heifers that are raised and sold often results in economic losses to producers when compared to the often high costs of rearing them. Increasing heifer survival can have significant economic benefits for dairy farms, as raising replacement heifers is a significant cost (VanRaden et al., 2021). However, alternatives can be implemented to reduce these costs, such as selling younger heifers, raising fewer spare heifers, or rearing part of the herd to beef semen. A compromise between genetic improvement and replacement rate must also be achieved.

Small differences in culling reasons were observed across the seasons, with fall and winter having the highest culling rates (27.30% and 26.65%, respectively), while spring and Summer had the lowest culling rates (23.72% and 22.33%, respectively). There are studies showing that the time of conception of the cow can influence the life of the progeny. Pinedo and De Vries (2017) observed a difference in the offspring’s survival and performance according to the time of conception, where Holstein cows that conceived in winter had better results than those cows that conceived in summer. This season effect may be related to thermal stress, which has influence on production, reproduction, health, placental development, and immunity of offspring (Tao and Dahl, 2013). Heat stress can affect progesterone levels in the blood with negative impact on reproduction (De Rensis and Scaramuzzi, 2003). Pinedo et al. (2010) reported an increase in culling rates due to reproductive reasons after the summer, probably because the cows did not get pregnant during that summer season.

This study also showed that the geographical location of the herd can influence the frequencies of the culling reasons. For example, in the two main dairy provinces in Canada, Quebec and Ontario, there is a predominance of cullings due to health and production reasons, respectively. The higher frequency of culling due to health in Quebec could be simply related to their health recording system, which is based on veterinary recording in contrast to producer recording in Ontario (Koeck et al., 2012). In addition, the government of Quebec has been adopting measures to enhance cattle health through the Animal Health Improvement Program in Quebec (ASAQ Agreement) (Domaine Bioalimentaire Québécois & Aviaire, 2021).

The effects of the climatic regions and ecozones were also significant and may be associated with changes in the physiology of dairy cows due to thermal stress. A study conducted in Germany indicated that the rate of the conception in dairy cows is affected by thermal stress (Schüller et al., 2014). In Quebec, Ouellet et al. (2019) reported that Holstein cows reared in the humid continental climate showed a reduction in protein and fat yields, but with little effect on milk production. In addition, Campos et al. (2022) investigated thermal stress effects on milk production traits in the provinces of Quebec and Ontario using meteorological data and found a significant decrease in milk, fat, and protein yields, which caused economic losses.

A large number of culling reasons are still unknown (Supplemental Table 1), highlighting the further need for improvement to the current recording system. It is essential to continue improving management strategies and selecting for greater longevity, which has economic and environmental implications for the dairy industry. When evaluating overall cow longevity and cow longevity based on the different culling reasons, factors such as year, province, season, climatic region, and ecozona should be considered, as they significantly affected the frequencies of culling reasons in Canadian Holsteins.

## CONCLUSIONS

The frequency of culling reasons in Canadian Holstein cattle has changed over time and differed based on year, cow age, province, climatic regions, and ecozones. The main culling reasons are reproductive issues, milk production, and health problems. The characterization of culling reasons indicates the need for continued refinement in the national selection indexes, with a greater emphasis on reproduction, health, and welfare traits to increase cow productive life.

## Supplementary Material

txad102_suppl_Supplementary_Material
