# Supplementary material for: A comprehensive characterization of longevity and culling reasons in Canadian Holstein cattle based on various systematic factors
Source: Transl Anim Sci. 2023 Aug 28;7(1):txad102. doi: 10.1093/tas/txad102 (PMC10576516; doi:10.1093/tas/txad102)
Supplement: txad102_suppl_Supplementary_Material [file txad102_suppl_supplementary_material.docx]

**Supplementary Table 1.** Annual distribution of frequencies of the eight culling reasons in Canadian Holstein cows.

| Year | Culling reasons | | | | | | | | Overall frequencies | |
| --- | --- | --- | --- | --- | --- | --- | --- | --- | --- | --- |
|  | Reproduction | Production | Health | Conformation | Economic | Accident | Age-related causes | Workability |  |  |
| 1996 | 494 | 706 | 506 | 448 | 13 | 68 | 54 | 40 | 2,329 | 0.10% |
| 1997 | 2,429 | 2,924 | 2,032 | 1,499 | 54 | 525 | 114 | 143 | 9,720 | 0.41% |
| 1998 | 2,535 | 3,293 | 2,567 | 1,792 | 422 | 614 | 148 | 130 | 11,501 | 0.48% |
| 1999 | 2,759 | 3,926 | 3,048 | 2,011 | 848 | 725 | 190 | 191 | 13,698 | 0.57% |
| 2000 | 3,225 | 6,371 | 4,423 | 3,088 | 2,711 | 1,163 | 217 | 531 | 21,729 | 0.91% |
| 2001 | 6,636 | 11,126 | 6,578 | 4,304 | 3,537 | 1,751 | 282 | 573 | 34,787 | 1.45% |
| 2002 | 10,627 | 15,396 | 9,446 | 6,512 | 5,595 | 2,569 | 339 | 744 | 51,228 | 2.14% |
| 2003 | 11,850 | 12,383 | 10,124 | 7,048 | 4,879 | 2,648 | 499 | 757 | 50,188 | 2.10% |
| 2004 | 16,194 | 12,573 | 13,305 | 9,606 | 6,732 | 3,013 | 737 | 1,030 | 63,190 | 2.64% |
| 2005 | 18,195 | 14,048 | 15,386 | 10,901 | 6,959 | 3,559 | 1,050 | 1,363 | 71,461 | 2.99% |
| 2006 | 20,846 | 16,243 | 17,834 | 13,012 | 6,991 | 4,587 | 1,290 | 1,914 | 82,717 | 3.46% |
| 2007 | 21,343 | 17,104 | 18,345 | 11,892 | 8,105 | 5,184 | 1,281 | 1,628 | 84,882 | 3.55% |
| 2008 | 23,444 | 22,477 | 19,996 | 12,871 | 10,416 | 5,808 | 1,288 | 1,562 | 97,862 | 4.09% |
| 2009 | 26,151 | 20,689 | 22,013 | 14,767 | 8,246 | 6,486 | 1,558 | 1,642 | 101,552 | 4.24% |
| 2010 | 31,182 | 24,294 | 29,529 | 19,310 | 7,965 | 7,777 | 1,905 | 2,355 | 124,317 | 5.20% |
| 2011 | 31,615 | 24,335 | 29,667 | 18,716 | 9,955 | 8,203 | 2,084 | 2,122 | 126,697 | 5.30% |
| 2012 | 36,061 | 27,319 | 34,717 | 22,454 | 12,221 | 9,337 | 2,452 | 2,617 | 147,178 | 6.15% |
| 2013 | 37,754 | 30,710 | 36,636 | 24,612 | 15,269 | 9,735 | 2,834 | 3,055 | 160,605 | 6.71% |
| 2014 | 37,662 | 30,709 | 33,004 | 21,809 | 17,304 | 9,356 | 2,769 | 2,713 | 155,326 | 6.49% |
| 2015 | 35,738 | 28,872 | 32,592 | 21,427 | 26,782 | 9,149 | 2,908 | 2,371 | 159,839 | 6.68% |
| 2016 | 34,818 | 29,501 | 31,177 | 20,728 | 24,818 | 9,066 | 2,908 | 2,333 | 155,349 | 6.49% |
| 2017 | 33,469 | 27,161 | 29,244 | 18,702 | 28,079 | 8,744 | 2,880 | 1,978 | 150,257 | 6.28% |
| 2018 | 37,413 | 33,827 | 31,345 | 21,421 | 45,439 | 9,032 | 3,505 | 2,371 | 184,353 | 7.70% |
| 2019 | 34,812 | 37,882 | 28,110 | 19,771 | 32,349 | 8,473 | 3,381 | 2,390 | 167,168 | 6.99% |
| 2020 | 33,589 | 44,208 | 26,310 | 18,971 | 27,847 | 8,211 | 3,281 | 2,377 | 164,794 | 6.89% |

**Supplementary Table 2*.*** Distribution of frequencies for the eight culling reasons by season in Canadian Holstein cows.

| Culling reasons | Season | | | |
| --- | --- | --- | --- | --- |
|  | Fall | Winter | Spring | Summer |
| Reproduction | 160,294 | 153,177 | 127,755 | 109,615 |
| Production | 137,691 | 137,357 | 120,832 | 102,197 |
| Health | 130,327 | 123,971 | 113,377 | 120,259 |
| Conformation | 90,823 | 87,115 | 76,132 | 73,602 |
| Economic | 77,895 | 79,240 | 76,965 | 79,436 |
| Accident | 35,117 | 34,724 | 32,937 | 33,005 |
| Age-related causes | 11,263 | 10,812 | 9,230 | 8,649 |
| Workability | 9,829 | 11,354 | 10,325 | 7,422 |
| Overall frequencies | 653,239 | 637,750 | 567,553 | 534,185 |
|  | 27.30% | 26.65% | 23.72% | 22.33% |

**Supplementary Table 3.** Distribution of frequencies for the eight culling reasons by province in Canadian Holstein cows.

| Culling reasons | Province | | | | | | | | | |
| --- | --- | --- | --- | --- | --- | --- | --- | --- | --- | --- |
|  | Ontario | Quebec | Alberta | British Columbia | Manitoba | Saskatchewan | New Brunswick | Nova Scotia | Prince Edward Island | Newfoundland and Labrador |
| Reproduction | 223,594 | 162,685 | 58,635 | 50,575 | 22,443 | 12,339 | 7,263 | 6,885 | 5,756 | 666 |
| Production | 263,767 | 80,985 | 52,758 | 49,898 | 16,596 | 13,850 | 6,138 | 7,035 | 6,463 | 587 |
| Health | 166,215 | 182,164 | 44,064 | 41,424 | 20,736 | 12,817 | 6,995 | 6,921 | 5,951 | 647 |
| Conformation | 118,406 | 100,926 | 39,683 | 31,372 | 15,452 | 9,274 | 4,388 | 4,506 | 3,351 | 314 |
| Economic | 113,762 | 100,025 | 33,567 | 29,729 | 13,282 | 10,969 | 4,448 | 4,324 | 3,315 | 115 |
| Accident | 36,154 | 64,785 | 9,227 | 11,201 | 4,301 | 3,412 | 2,632 | 2,458 | 1,413 | 200 |
| Age-related causes | 16,936 | 10,227 | 4,898 | 3,804 | 1,678 | 1,116 | 500 | 322 | 429 | 44 |
| Workability | 12,946 | 12,642 | 5,558 | 3,212 | 2,114 | 1,156 | 492 | 433 | 310 | 67 |
| Overall frequencies | 951,780 | 714,439 | 248,390 | 221,215 | 96,602 | 64,933 | 32,856 | 32,884 | 26,988 | 2,640 |
|  | 39.78% | 29.86% | 10.38% | 9.25% | 4.04% | 2.71% | 1.37% | 1.37% | 1.13% | 0.11% |

**Supplementary Table 4.** Distribution of frequencies for the eight culling reasons by climatic regions in Canadian Holstein cattle.

| Culling reasons | Climate | | | | | | |
| --- | --- | --- | --- | --- | --- | --- | --- |
|  | BSk | Cfb | Csb | Dfa | Dfb | Dfc | ET |
| Reproduction | 6,045 | 34,573 | 5,417 | 101 | 485,614 | 18,881 | 210 |
| Production | 6,798 | 31,842 | 6,889 | 28 | 437,876 | 14,449 | 195 |
| Health | 6,150 | 30,051 | 3,409 | 73 | 431,974 | 16,195 | 82 |
| Conformation | 5,546 | 21,754 | 3,376 | 33 | 286,338 | 10,541 | 84 |
| Economic | 3,910 | 21,487 | 2,584 | 18 | 274,132 | 11,374 | 31 |
| Accident | 1,718 | 8,241 | 908 | 9 | 120,720 | 4,148 | 39 |
| Age-related causes | 680 | 2,721 | 432 | 10 | 34,823 | 1,264 | 24 |
| Workability | 677 | 2,066 | 404 | 8 | 34,278 | 1,485 | 12 |
| Overall frequencies | 31,524 | 152,735 | 23,419 | 280 | 2,105,755 | 78,337 | 677 |
|  | 1.32% | 6.38% | 0.98% | 0.01% | 88.01% | 3.27% | 0.03% |

BSk = Middle Latitude Steppe climate (Cold semi-arid), Cfb = Marine West Coast climate (Oceanic), Csb = Subtropical or Mediterranean climate (dry and cool summer), Dfa = Humid and hot summer Continental climate, Dfb = Humid and cool summer Continental climate, Dfc = Subarctic climate, and ET = Tundra climate

**Supplementary Table 5.** Frequencies of eight culling reasons by ecozones in Canadian Holstein cows

| Culling reasons | Ecozone | | | | | | |
| --- | --- | --- | --- | --- | --- | --- | --- |
|  | MixedWood Plain | Atlantic Maritime | Prairie | Pacific Maritime | Boreal Shield | Boreal Plain | Montane Cordillera |
| Reproduction | 312,974 | 69,744 | 77,888 | 40,111 | 24,698 | 15,019 | 10,407 |
| Production | 306,641 | 44,136 | 71,397 | 38,916 | 14,548 | 11,524 | 10,915 |
| Health | 267,642 | 74,503 | 66,046 | 33,514 | 27,158 | 11,220 | 7,851 |
| Conformation | 174,733 | 43,528 | 55,545 | 25,168 | 13,977 | 8,550 | 6,171 |
| Economic | 164,995 | 45,101 | 47,370 | 24,356 | 16,074 | 10,337 | 5,303 |
| Accident | 72,943 | 26,576 | 15,029 | 9,162 | 8,247 | 1,798 | 2,028 |
| Age-related causes | 22,288 | 4,613 | 6,534 | 3,160 | 1,609 | 1,109 | 641 |
| Workability | 19,610 | 5,541 | 7,514 | 2,480 | 1,803 | 1,253 | 729 |
| Overall frequencies | 1,341,826 | 313,742 | 347,323 | 176,867 | 108,114 | 60,810 | 44,045 |
|  | 56.08% | 13.11% | 14.52% | 7.39% | 4.52% | 2.54% | 1.84% |
